# Robust spinal neuroinflammation mediates mechanical allodynia in Walker 256 induced bone cancer rats

**DOI:** 10.1186/1756-6606-5-16

**Published:** 2012-05-20

**Authors:** Qi-Liang Mao-Ying, Xiao-Wei Wang, Chang-Jiang Yang, Xiu Li, Wen-Li Mi, Gen-Cheng Wu, Yan-Qing Wang

**Affiliations:** 1 Department of Integrative Medicine and Neurobiology, Shanghai Medical College, Institute of Acupuncture Research, Institutes of Brain Science, State Key Laboratory of Medical Neurobiology, Fudan University, Shanghai, China

**Keywords:** Cancer induced pain, Glial activation, Toll-like receptor 4, Neuroinflammation, Rat

## Abstract

It has been reported that remarkable and sustained activation of astrocytes and/or microglia occurs in cancer induced pain (CIP), which is different from neuropathic and inflammatory pain. The present study was designed to investigate the role of spinal Toll-like receptor 4 (TLR4) induced glial neuroinflammation in cancer induced pain using a modified rat model of bone cancer. The rat model of CIP consisted of unilateral intra-tibial injection with Walker 256 mammary gland carcinoma. Nine days after Walker 256 inoculation, a robust activation of both astrocytes and microglia in bilateral spinal dorsal horn was observed together with significant bilateral mechanical allodynia. This neuroinflammation was characterized by enhanced immunostaining of both glial fibrillary acidic protein (GFAP, astrocyte marker) and OX-42 (microglia marker), and an elevated level of IL-1β, IL-6 and TNF-α mRNA. I.t. administration of fluorocitrate (an inhibitor of glial metabolism, 1 nmol) or minocycline (an inhibitor of microglia, 100 μg) has significant anti-allodynic effects on day 12 after Walker 256 inoculation. Naloxone (a nonstereoselective TLR4 signaling blocker, 60 μg, i.t.) also significantly alleviated mechanical allodynia and simultaneously blocked the increased inflammatory cytokine mRNA. The results suggested that spinal TLR4 might play an important role in the sustained glial activation that critically contributed to the robust and sustained spinal neuroinflammation in CIP. This result could potentially help clinicians and researchers to better understand the mechanism of complicated cancer pain.

## Introduction

Cancer induced pain (CIP), especially derived from primary bone cancer or secondary bone metastases, is a complicated clinical syndrome, which severely impairs the quality of life of patients. Due to the lack of elucidation of its mechanism, it still remains a serious medical problem. In the past decades, several experimental models have been established which shed some light on the mechanisms underlying CIP [1-4].

A remarkable and sustained activation of astrocyte and/or microglia induced by unilateral tumor cell inoculation has been reported in cancer induced pain models [1,5]. Especially in peripheral neuropathic pain and inflammatory pain models, former research suggested that glial activation and the subsequent robustly increased expression of proinflammatory cytokines critically contribute to chronic pain [6].

Recently, we established a new model of CIP by inoculation of mammary gland carcinoma cells Walker 256 into unilateral tibial cavity and observed bilateral mechanical allodynia [7]. Whether the above cellular and molecular changes indicating a contribution of neuroinflammation are associated with CIP remains to be elucidated in the present study.

There are numerous candidate neuron-to-glia signals proposed for initiating glial activation, including neurotransmitters, neuromodulators and neuronally derived chemokines among others [8]. A very intriguing mechanism has been recently proposed for spinal microglial activation in response to peripheral nerve injury, that is, activation of Toll-like receptors (TLRs) especially TLR4, TLR3 and TLR2 [9-13]. It has been reported that TLR4 is expressed on central nervous system (CNS) glia [14,15]. TLRs are Type I membrane receptors which recognize a variety of molecules derived from pathogens, such as bacterial cell wall components [16-19]. In addition, TLRs detect specific endogenous ligands, mainly molecules released from damaged cells or extracellular matrix breakdown products, which activate the innate immune system, including fibrinogen, heat shock proteins (HSP), mRNA, fibronectin, tenascin-C and so on [20-23]. However, the receptor leading to glial activation in chronic pain models and especially in CIP still remain unidentified.

Hence, the present study was designed to characterize the role of microglia and astrocyte activation dependent spinal neuroinflammation in a rat model of CIP. The results demonstrated that bilateral sustained activation of spinal astrocyte as well as microglia critically contributes to the bilateral mechanical allodynia induced by unilateral bone cancer in rats. Spinal TLR 4 might play an important role in the sustained activation of microglial cells in CIP rats.

## Results

### Bone destruction evaluation

**Figure 1:**
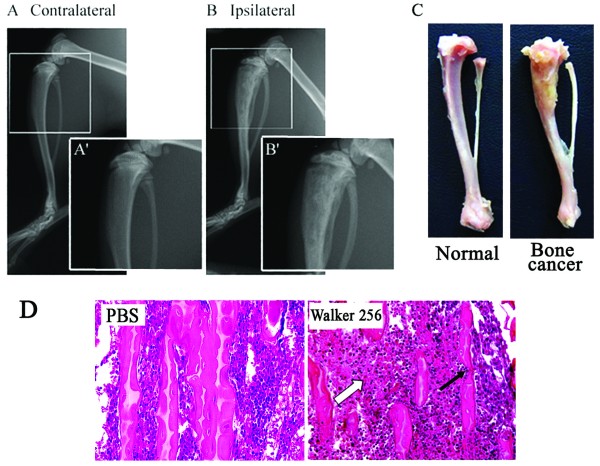
**Tibia bone destruction by Walker 256 inoculation.** Radiographs of the Contralateral (**A**) and Ipsilateral (**B**) tibia bone 20 days after Walker 256 inoculation. (**A’**-**B’**) showing the proximal end of the bones with a higher magnification. (**C**) showing the tibia bone from normal and tumor bearing rats 20 days after Walker 256 inoculation. (**D**) histology of tibial bone destruction stained by Hematoxylin-eosin. The hollow arrow showing normal marrow cavity in PBS rats instead tumor cells in Walker 256 inoculated rats. The black arrow indicates the trabecular bone.

At day 20 after Walker 256 inoculation, radiological photographs showed that the Walker 256 inoculated tibia bone displayed significant signs of radiolucent lesion in the proximal epiphysis, close to the inoculated site (Figure 1A, B). However, no radiological changes were observed in the tibia bone contralateral to Walker 256 inoculation. At the end of the experiment, rats were sacrificed with an overdose of sodium pentobarbital and the tibia bones were removed for histological analysis. The Walker 256 inoculated tibia bone showed significant tumor growth (Figure 1D) and the surface of bone was roughened in comparison to the normal tibia (Figure 1C).

### Time course of pain-related behaviors induced by Walker 256 inoculation

**Figure 2:**
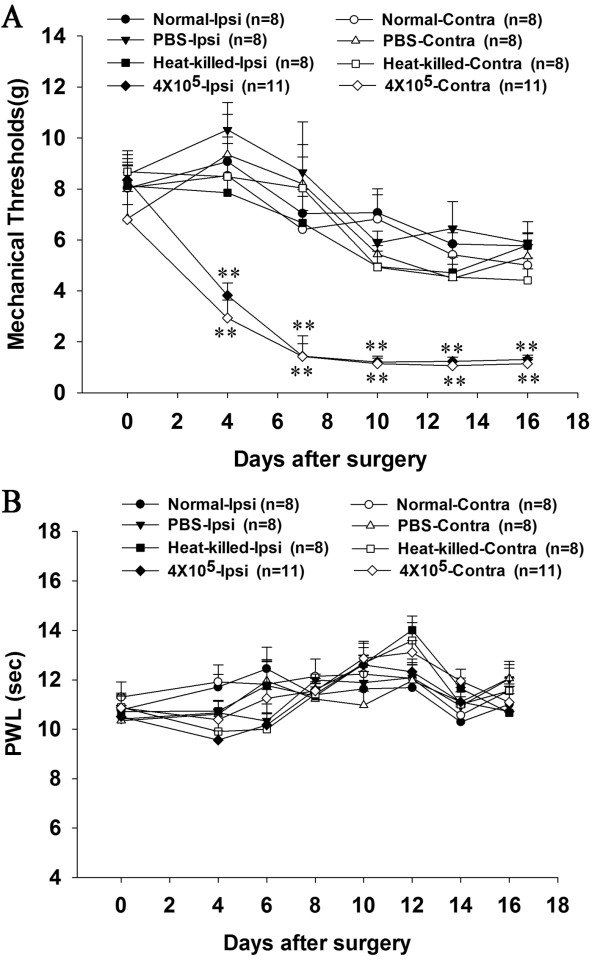
**Changes of mechanical response thresholds to von Frey hair (A) and paw withdrawal latency to radiant heat (B) of the ipsilateral and contralateral hind paw in normal rats and Walker 256 (4 × 10**^**5**^**cells), heat-killed cells and PBS -inoculated rats.** Data are expressed as means ± SEM. *p < 0.05, **p <0.01 vs. normal rats.

At 4 days after unilateral Walker 256 inoculation into the tibia bone, the rats displayed a profound decrease in paw withdrawal threshold to von Frey hair stimulation. This was not only observed in the paw ipsilateral to the Walker 256 inoculated hind limb but also in the contralateral hind limb that was not inoculated (p < 0.01). In contrast, there was no significant difference in paw withdrawal threshold between rats receiving an intra-tibial injection of PBS or heat-killed Walker 256 cells, and normal rats (p > 0.05) (Figure 2A). Using a radiant heat test, we showed that there were no detectable differences in thermal hyperalgesia between normal, PBS, heat-killed Walker 256, and Walker 256 inoculated rats for both hind paws during the whole experiment (p > 0.05) (Figure 2B).

### Hypertrophy of astrocytes and microglia in the spinal cord

**Figure 3:**
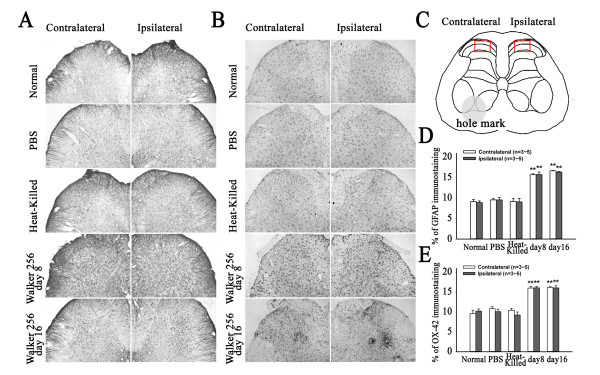
**Changes of expression of GFAP and OX-42 protein in the dorsal horn of the spinal cord detected by immunohistochemistry after the rats received carcinoma cells.** (**A**) and (**B**) showing representative images for GFAP and OX-42 immunostaining respectively. (**C**) showing the selected area marked with red square in the spinal cord for quantification. (**D**, **E**) Quantification of astrocyte and microglia expression in the spinal dorsal horn after intra-tibial inoculation of Walker 256. Percentages of immunostaining are expressed as mean ± SEM (3 ~ 5 for each group). **p <0.01 vs. normal rats.

Immunohistochemical staining of GFAP and OX-42 was used to evaluate the hypertrophy of astrocytes and microglia in the spinal cord respectively. The average of four sections per rats was analyzed in this study. The immunohistochemical results showed that in control rats, sporadic astrocyte moderately labeled by GFAP with classic star-shape, and microglia labeled by OX-42 with classic ramified morphology, antler-like fine processes and slender cytoplasm, are detected in both the gray and white matters of the spinal cord (Figure 3A-B). In the Walker 256 inoculated rats, a robust hypertrophy of both astrocyte and microglia was observed in both sides of spinal dorsal horn at 8 and 16 days after unilateral inoculation as compared to the normal, PBS, and heat-killed Walker 256 cells inoculated rats. The hypertrophic astrocytes and microglia turned to large cell shapes with short and thick processes. Quantification by an observer blinded for treatment showed that GFAP- and OX-42-positive signals in both sides of the spinal dorsal horn of Walker 256 inoculated rats were remarkably elevated in comparison to normal, PBS and heat-killed cells inoculated rats (Figure 3D, E).

### Changes in IL-1β, IL-6 and TNF-α mRNA expression in the spinal cord

**Figure 4:**
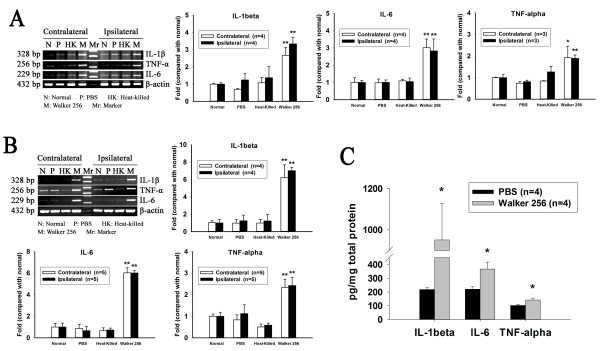
**Changes of inflammatory cytokines IL-1β, IL-6, TNF-α mRNA level detected by Semiquantitative RT-PCR analysis.** (**A**) and (**B**) showing the mRNA levels in spinal dorsal horn 8 days and 16 days after intra-tibial inoculation of Walker 256 respectively. The relative mRNA level was expressed as a ratio relative to the normal control. *p < 0.05, **p < 0.01 vs. normal rats. (**C**) showing the protein level of IL-1β, IL-6, TNF-α in the spinal dorsal horn at 12 days after Walker 256 inoculation. * p < 0.05, vs. PBS rats.

To determine the functional activation of spinal glia, the expression of mRNA for the proinflammatory cytokines IL-1β, IL-6 and TNF-α in the spinal dorsal horn was analyzed using semi-quantitative RT-PCR. The results showed that, at 8 days post-inoculation, the levels of IL-1β, IL-6 and TNF-α mRNA in both sides of spinal dorsal horn were elevated significantly in Walker 256 inoculated rats (p < 0.05). No change in cytokine mRNA was detected when comparing rats inoculated with PBS and Heat-killed cells with normal rats (p > 0.05) (Figure 4A). Furthermore, the levels of IL-1β, IL-6 and TNF-α mRNA were further elevated at 16 post-inoculation in comparison to the normal rats (p < 0.05) (Figure 4B). Similarly, the spinal level of IL-1β, IL-6 and TNF-α protein in Walker 256 inoculated rats were significantly increased at 12 days post Walker 256 inoculation (p < 0.05) (Figure 4C). Therefore, in order to minimize the number of animals needed, we only analyzed cytokine mRNA levels in the spinal in subsequent experiments.

### Role of spinal microglia activation in cancer induced pain

**Figure 5:**
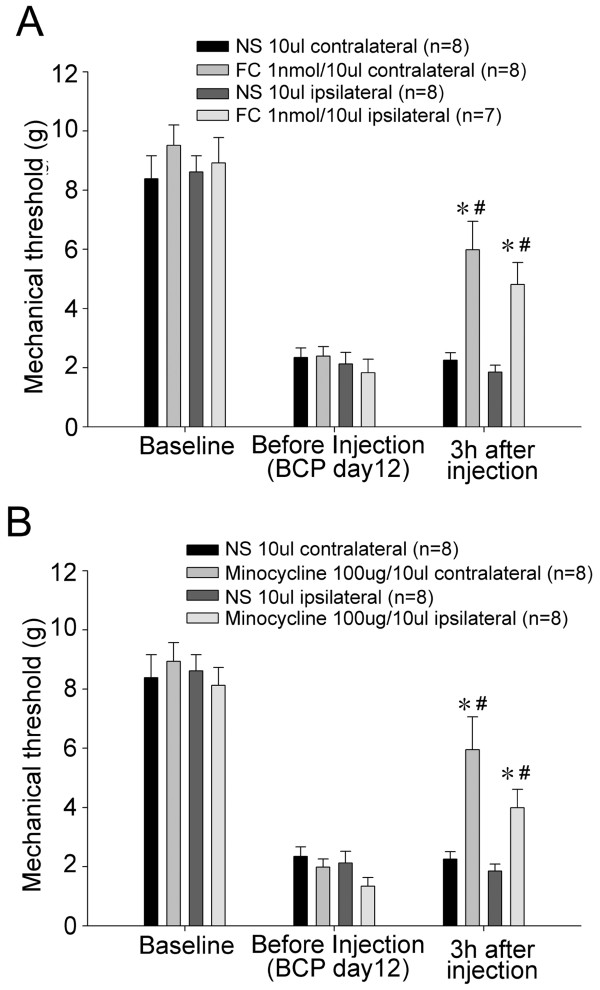
**Anti-allodynic effects by intrathecal administration of fluorocitrate (A) or minocycline (B).** On 12 days after Walker 256 inoculation, fluorocitrate (1 nmol), a glia metabolic inhibitor, or minocycline (100 μg), a kind of microglia activation inhibitor significantly increased the mechanical thresholds to von Frey hair stimulation 3 hrs after i.t. treatment. *p < 0.05 vs. normal saline treatment; #p < 0.05 vs. before treatment.

On day 12 after Walker 256 inoculation, the rats displayed stable mechanical allodynia, and also spinal astrocytes and microglia were observed to be significantly activated. Hence, to investigate the functional role of spinal glial hypertrophy in the present rat model of bone cancer pain, we examined behavioral pain responses after i.t. treatment with inhibitors of glial activity on day 12 after Walker 256 inoculation. The results showed that i.t. injection of 1 nmol fluorocitrate (a glia metabolic inhibitor) or 100 μg minocycline (an inhibitor of microglial activity) both significantly elevated mechanical thresholds in both hind paws (p < 0.05) (Figure 5A, B). A single i.t. administration of 10 μl normal saline or PBS/HCl as a control had no effect on mechanical thresholds in Walker 256 inoculated rats as compared to mechanical thresholds before intrathecal administration (p > 0.05).

### Role of Toll-like receptor 4 in cancer induced pain

**Figure 6:**
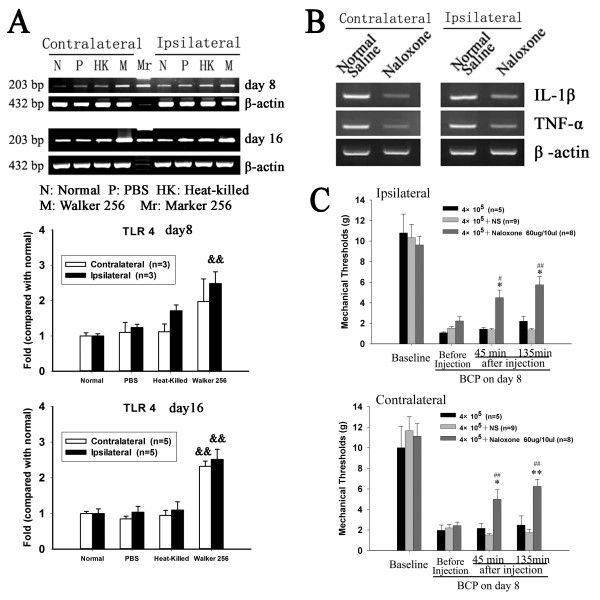
**Spinal Toll-like receptor (TLR4) is involved in bone cancer pain.** (**A**) showing the changes of TLR4 mRNA levels in bilateral dorsal horn of spinal cord 8 and 16 days after intra-tibial injection of PBS, Heat-killed or live Walker 256 cells. (**B**) Showing the blocking effect of single intrathecal administration of naloxone (60 μg) on elevated spinal IL-1β and TNF-α mRNA expression induced by intra-tibial inoculation of Walker 256 cells. (**C**) Anti-allodynic effects induced by single intrathecal administration of naloxone (60 μg). On the day 8 after Walker 256 inoculation, naloxone (60 μg), a nonsteroselective TLR4 antagonist, significantly increased the mechanical thresholds to von Frey hair stimulation 45, 135 mins after i.t. treatment. &&p < 0.01 vs. normal rats; *p < 0.05, **p < 0.01 vs. Walker 256 inoculated rats; #p < 0.05, ##p < 0.01 vs. normal saline treatment.

A possible mechanism contributing to spinal glial activation is via stimulation of TLR4, a member of the TLR family. It has been proposed that sensory neuron damage leads to the release of TLR recognized signals and consequently to stimulation of microglial TLR4, initiating microglial activation with release of proinflammatory cytokines. RT-PCR analysis showed that the levels of TLR4 in bilateral spinal dorsal horn were significantly increased after unilateral inoculation of Walker 256 in comparison to normal rats or rats inoculated with PBS or heat-killed cells. Notably, the levels of TLR4 were increased moderately at 8 days and markedly at 16 days after Walker 256 inoculation (Figure 6A). Based on previous studies showing that naloxone effectively inhibits the TLR4 signaling pathway [15,24,25], 60 μg of naloxone was administered through i.t. injection. The behavioral test showed that, as compared to the i.t. normal saline injection, a single i.t. administration with naloxone significantly increased the mechanical thresholds of both hind paws at 45 and 135 mins after intrathecal injection, on 8 days after Walker 256 inoculation (Figure 6C). Further RT-PCR analysis showed that i.t. naloxone treatment significantly decreased spinal IL-1β and TNF-α mRNA bilaterally 135 mins after intrathecal injection as compared to rats treated with saline (Figure 6B).

## Discussion

The main finding of the present study is the sustained and bilateral activation of both spinal astrocyte and microglia in response to unilateral Walker 256-injection into the tibia of rats. Moreover, the inhibition of glial activation by i.t. injection of fluorocitrate or minocycline both reduced the mechanical allodynia in rats with CIP. These findings clearly demonstrate that spinal glial activation and sustained spinal neuroinflammation contribute to the development and maintenance of allodynia in cancer induced pain. Further studies suggested that the increased spinal TLR4 expression might play an important role in the sustained glial activation we observed in our model of CIP.

According to a previously reported rat CIP model [2], we established a modified rat model of cancer induced pain by inoculating an alternative and available breast cancer cell line, Walker 256 mammary gland carcinoma cells, into the intra-tibial cavity of syngeneic female Wistar rats via a new injection site [7]. In the present study, no skin incision was made along the patellar ligament to expose the tibia head and the carcinoma cells were injected directly into the tibial cavity via knee joint. This modification of the inoculation procedure was made carefully to further minimize the damage to the knee joint. The results demonstrated that the inoculation procedure per se did not affect basal behavioral responses in the von Frey and radiant heat test even on the early days after inoculation, indicating that the function of the knee joints was kept intact. However, our preliminary results indicate that the surgical procedure may affect the general condition at the early stage, showing the significant body weight loss at early days after intra-tibial injection, even in PBS injected rats. Therefore, we selected a later time point (as early as the pain became stable) to analyze glia activation and TLR4 expression.

Clinically, the severity of bone cancer induced pain, which is usually induced by primary bone cancer or by secondary bone metastases from e.g. breast, prostate or lung cancer is closely correlated with the extent of bone destruction. The pain progressively becomes heavy as cancer growth and bone destruction in the affected bone [26,27]. The present CIP model, using an intra-tibial Walker 256 cell inoculation, is associated with ambulatory pain, mechanical allodynia and a reduction in weight bearing on the ipsilateral hind limb indicating the hypersensitivity of rats to pain [7]. Similar to most other reported CIP models, thermal hyperalgesia was not observed after Walker 256 cells inoculation. The present results suggested that this modified model could also mimic well the key features of human CIP.

Interestingly, in the present model of cancer induced pain, the rats displayed mechanical allodynia in both hind paws even though Walker 256 cells were inoculated only unilaterally. These findings demonstrate the existence of bilateral pain-related behavior. Furthermore, we observed a bilateral significant increase in the expression of GFAP and OX-42 as well as proinflammatory cytokines including TNF-α, IL-β, IL-6 mRNA. These observations indicate bilateral activation of both microglia and astrocytes in the present bone cancer pain model. This unique sustained bilateral activation of both microglia and astrocytes in the present model of CIP is quite different from what is observed in other cancer pain models as well as in unilateral models of neuropathic pain and inflammatory pain. In osteolytic sarcoma cell- and mammary gland carcinoma cell-induced bone pain models [1,2,28], remarkable and sustained activation of astrocytes only was observed ipsilateral to cancer cells inoculation. However, a sustained ipsilateral activation of both astrocyte and microglia 20 days after the inoculation of prostate cancer cells was reported later [5]. This apparent discrepancy illustrates the complexity of the neuroinflammatory condition in the spinal cord induced by different cancer cells in different animals and these findings further remind us of the complexity of the underlying mechanisms of cancer pain.

An early activation of spinal microglia and a delayed activation of astrocytes, which in turn maintains long-term pathological states such as persistent pain, have been observed after peripheral nerve injury [29]. Hald et al. compared the differential activation of spinal glial cells in two murine models of cancer pain and one model of neuropathic pain to investigate if microglial activation is a general prerequisite for astrocyte activation in pain models. These authors found that bone cancer and sciatic nerve proximal cancer (developing from NCTC-2472 cells) induced pain resulted in severe spinal astrogliosis without activation of microglia, while sciatic nerve injury led to a transient activation of microglia and sustained astrogliosis [28]. These observations might indicate that development of hypersensitivity and astrocyte activation in CIP models could take place independently of microglial activation. Actually, the activation of microglia does not appear earlier than activation of astrocyte in this and another reported CIP model [5]. However, in the present study, minocycline (an inhibitor of microglia) reduced the allodynia during the middle stage (day 12) of cancer pain which demonstrated that microglia also plays an essential role in the maintenance of sustained mechanical allodynia in the present CIP model. The results further implied a unique sequence of glial activation in the spinal cord in this CIP model.

Excitation of glia at one site can activate distant glia through gap junctions and propagated calcium waves, causing them to release pain-enhancing substances such as proinflammatory cytokines, glutamate, nitric oxide, or other products as well [30,31]. Therefore, the widespread activation of glia in spinal cord was proved to be involved in the mirror-image pain induced by neuropathy [32]. Actually, glial cells are well characterized for mediating expansions of the body region from which pain is perceived [33]. Once the spread of excitation reaches the contralateral dorsal horn, mirror-image pain would occur. Indeed Milligan et al. have demonstrated that inhibiting the function of astrocyte gap junctions could abolish sciatic inflammatory neuropathy induced mirror-image pain [32]. In the present study, contralateral spreading of both microglia and astrocyte activation was observed in the spinal cord of rats with bilateral allodynia induced by unilateral bone cancer. Moreover, inhibiting glial activation produced bilateral attenuation of allodynia. These results further indicate the important role of glial activation in chronic pain.

Previous in vivo and in vitro studies have established that in the CNS, TLR4 is almost exclusively expressed by microglia [34,35]. In this study, upregulation of TLR4 mRNA in bilateral spinal dorsal horn at 8 and 16 days after cancer cell-inoculation was observed. Our preliminary data from flow cytometric analysis showed that TLR4 expressed on microglia surface was increased from 4.1% in normal rats to 5.6% Walker 256 inoculated rats (data not shown). It has been reported that naloxone can effectively inhibit microglial activation and the production of TNF-α and IL-1β in response to activation of the TLR signaling pathway [25]. In addition, naloxone is now widely used as nonstereoselective TLR4 signaling pathway blocker in the study of the TLR4 [15,24,36]. Hutchinson et al. reported that i.t. delivery of neuronally inactive naloxone (60 μg), a novel toll-like receptor (TLR) 4 antagonist, can reverse the chronic constriction injury (CCI)-induced neuropathic pain in rats. Further histochemical analysis showed that sustained i.t. delivery of neuronally inactive naloxone produced suppression of CD11b/c while they showed no effect on GFAP expression in rats with CCI induced neuropathic pain. Interestingly, i.t. naloxone significantly attenuated the bilateral allodynia and blocked the increased level of spinal TNF-α and IL-1β mRNA induced by CIP, indicating that TLR4 might contribute to the microglia activation in the present study. This is very consistent with the results from previous studies [25,36-38]. Based on the pain relieving effect and reversal of cytokines production by naloxone and the previous reports [24,25,36], we propose that the analgesic effect is through glial inactivation by its action as a TLR4 antagonist. Moreover, our recent study showed that TLR2 expression were also elevated in spinal cord in CIP rats (data not shown), indicating a possible role of TLR2 and even other TLRs in cancer induced pain similar to what has been observed in neuropathic pain [18,39,40]. To date, very few endogenous ligand(s) that initiate the TLR4 signaling pathway in the CNS post-nerve injury or cancer development have been identified [41,42]. Further studies searching for the endogenous ligands for TLRs in cancer induced pain will shed light on finding new therapeutic targets for controlling cancer induced pain.

## Conclusion

The present study revealed a complicated condition of neuroinflammation in spinal cord with sustained, robust and bilateral activation of both microglia and astrocytes, which critically contributed to the maintenance of the bilateral allodynia in CIP. Our findings also indicate that the spinal TLR4 signaling pathway might play an important role in the observed sustained glial activation.

## Material and methods

### Animals

Female Wistar rats weighing 140-160 g were purchased from Shanghai Laboratory Animal Center, Chinese Academy Sciences. Rats were housed in temperature-controlled (24 ± 0.5°C) and light-controlled (12:12 h alternating light-dark cycle) room with free access to food and water. All experimental procedures were approved by the Animal Care and Use Committee (ACUC) of Fudan University, and were consistent with the NIH’s Guide for the Care and Use of Laboratory Animals and the Ethical Issues of the IASP [43].

### Inducement for cancer induced pain

According to the previous reports [7,44], the procedure for the inducement of caner induced pain was as follows. Briefly, Walker 256 rat mammary gland carcinoma cells were collected from cancerous ascitic fluid (derived from Wistar rat, Shanghai Institute of Biomedical Engineering). The cells were washed with PBS, counted using a haemocytometer and diluted to a final concentration of 1 × 10^8^ cells/ml PBS solution. For the sham group, heat-killed Walker 256 cells were prepared in the same final concentrations for injection and boiled for 5 min. The rats were anesthetized with sodium pentobarbital (50 mg/kg, i.p.) for surgery. Four microliter of carcinoma cells (4 × 10^5^), heat-killed carcinoma cells (sham group) in 4 μl of PBS, or 4 μl of PBS only (vehicle group) were slowly injected into the tibia cavity by using a 10 μl microinjection syringe with 23-gauge needle. The syringe was left in place for an additional 2 min to prevent the carcinoma cells from leaking out along the injection track. All animals were allowed to recover from the surgery for 3 days prior to any experimentation.

### Bone radiological detection and bone histology

Consistent with previous reports [7], to assess the tibia bone destruction by tumor, rats were placed on a clear plane plexiglass, exposed to an X-ray source under sodium pentobarbital anesthesia on day 20 after Walker 256 inoculation, and tibia bone radiographs were performed using E-COM Digital Radiographer System (E-COM Technology Co. LTD., Guangdong, China).

For bone histology, rats were transcardially perfused with 300 ml of 0.9% normal saline followed with 300 ml 4% paraformaldehyde after being anesthetized with an overdose of sodium pentobarbital. Tibia bones were removed and decalcified in decalcifying solution for 24 h. The bones were rinsed, dehydrated, and then embedded in paraffin, cut into 7 μm cross-sections using a rotary microtome (Reichert-Jung 820, Cambridge Instruments GmbH, Germany), and stained with hematoxylin and eosin to visualize the extent of tumor infiltration and bone destruction.

### Von Frey test for mechanical allodynia

Mechanical allodynia was measured as the hind paw withdrawal response to von Frey hair stimulation according to the up-down method [45]. Animals were placed in a plastic cage (26 × 20 × 14 cm^3^) with a plexiglass floor (containing 1.5 mm diameter holes in a 5 mm grid of perpendicular rows). Testing was performed by an observer blind with respect to group. After 15 min accommodation, an ascending series of von Frey hairs (0.40, 0.60, 1.4, 2.0, 4.0, 6.0, 8.0 and 15.0 g) (Stoelting, Wood Dale, Illinois, USA) were applied perpendicular to the mid-plantar surface of hind paw. Each hair was held about 1 ~ 2 s, with a 10-min interval. A trial began with the application of the 2.0 g hair. A positive response was defined as a withdrawal of hind paw upon the stimulus. Whenever a positive response to a stimulus occurred, the next lower hair was applied, and whenever a negative response occurred, the next higher hair was applied. The testing consisted of five more stimuli after the first change in response occurred, and the pattern of response was converted to a 50% von Frey threshold using the method described previously [45].

### Hargreaves’ test for thermal hyperalgesia

The paw withdrawal latency (PWL) to radiant heat was examined for evidence of heat hyperalgesia using the Model 390 Paw Stimulator Analgesia Meter (IITC/Life Science Instruments, USA) [46]. The rats were placed beneath an inverted, clear plastic cage upon an elevated floor of window glass. After 30 min adaptation, radiant heat was applied to the plantar surface of each paw until the animal lifted its paw from the glass. The time from onset of radiant heat application to withdrawal of the rat’s hind paw was defined as the PWL. The radiant heat was adjusted at a certain constant intensity to elicit the PWL around 10 ~ 12 s in normal rats. A cut-off time was defined 20 s to prevent tissue damage. Both hind paws were tested independently with a 10-min interval. The average of the three trials was then determined. Testing was performed blind with respect to group.

### Intrathecal drug administration

In order to observe the role of spinal astrocyte and microglia in CIP rats, fluorocitrate (FC, 1 nmol in 10 μl; Sigma) or minocycline hydrochloride (100 μg in 10 μl; Sigma) was administrated via lumbar puncture [47,48]. Fluorocitrate, a reversible glial metabolic inhibitor, acts by inhibiting aconitase, an enzyme of the Krebs energy cycle of glia but not neurons. Fluorocitrate was dissolved initially in 2 M HCl and then diluted in sterile 10 mM PBS to attain a final concentration, pH 6.0 at the time of testing. Minocycline hydrochloride, a second-generation semi-synthetic tetracycline derivative, was used to inhibit microglia activation. Minocycline hydrochloride was dissolved in saline freshly before use and heated briefly in a water bath until completely dissolved into the clear solution, then neutralized to pH (7.2-7.4) by 0.1 N NaOH. Based on previous studies showing that naloxone could block the TLR4 signaling pathway [14,15,24,49], naloxone (Sigma, St. Louis, USA) was intrathecally administered as a non-stereoselective TLR4 signaling blocker to determine the role of TLR4 on CIP in this study. During lumbar puncture [50], the rats were anesthetized with 2% isoflurane in air via a nose cone. After the lumbar region was disinfected with 70% v/v ethanol, a 29-gauge microinjection syringe needle filled with 10 μl drug was inserted via L5-6 interspace. The correct subarachnoid positioning of the tip of the needle was verified by a tail- or paw-flick response.

### Immunohistochemistry

Three to five animals of each group were used for immunohistochemical study. The rats were given an overdose of sodium pentobarbital and transcardially perfused with 300 ml 0.9% normal saline followed with 300 ml 4% paraformaldehyde in 0.1 M phosphate buffer (PB, pH 7.4). The L4-6 segments of spinal cord were removed, and then immersed from 10-30% gradient sucrose in PB for 24-48 h at 4°C. Transverse spinal sections (Free-floating, 25 μm) were cut in a cryostat (Reichert-Jung, German) for immunohistochemistry analysis. In order to distinguish the two sides of spinal cord, a thin needle was used to drill a hole in the contralateral side of the spinal cord before cutting on a freezing microtome. Sections were rinsed in 0.01 M PBS (pH 7.4) three times for 15 minutes, preincubated with 10% normal goat serum in 0.01 M PBS with 0.3% Triton-X-100 for 2 h at room temperature (RT), incubated over night at 4°C after 1 h at RT with mouse anti-glial fibrillary acidic protein (GFAP, 1:1000, Lab Vision) or mouse anti-OX-42 (1:1000, Chemicon) primary antibody. The sections were washed three times in 0.01 M PBS and incubated for 2 h at RT with biotinylated goat anti-mouse IgG (1:200, Santa Cruz). Then, the sections were washed three times in 0.01 M PBS and incubated for 1 h with avidin-biotin-peroxidase complex (1:200, Vector Laboratories) at RT. 0.05% 3,3-diaminobenzidine (DAB, Sigma) and 0.03% H_2_O_2_ were used to reveal the presence of the GFAP- or OX-42-positive signals following washed three times in PBS. The sections were then mounted, air-dried, dehydrated in alcohol in a graded manner, cleared in xylenes and cover slipped. To assess staining specificity, the primary antibody was omitted as negative control and showed no signs of immunohistochemical reaction. The stained sections were examined with a Leica Q500IW image analysis system. For GFAP or OX-42 immunostaining, positively labeled identified for automated counting using Image Measurement 1.0. Quantification of GFAP or OX-42 immunoreactivity was performed by calculating the percentages of immunostaining ([number of pixels of positively labeled objects within the fixed area]/[number of pixels within the same fixed area] × 100) [51]. Labeling was quantified in the selected area of spinal cord slices stained with diaminobenzidine, with four slices quantified per animal. The investigator responsible for image analysis was blind to the experimental condition of each rat.

### RT-PCR analysis

Three or five animals of each group were used for RT-PCR analysis. Total RNA was extracted from L4-6 segments of spinal cord by using the Trizol reagent (Invitrogen, CA, USA) according to manufacture’s instruction. The quality of RNA was assessed by agarose gel electrophoresis. 1 μg of total RNA was reversely transcribed into cDNA for RT-PCR. Negative control reactions were run without RNA to test for contamination of reagents. Oligonucleotide primers were chosen on the basis of rat nucleotide sequences in the GenBank database, and according to previously published sequences [52,53]. The size and sequence of each primer and number of cycles used are given in Table 1.

**Table 1:** Sequences of the forward and reverse primers and PCR conditions used for RT-PCR

**GenBank accession**	**Target gene**	**Primers**	**PCR condition (temperature/time)**			**Predicted size (bp)**
			**Denature**	**Anneal**	**Extend**	
NM031144	β-actin	Forward: 5’-tcaggtcatcactatcggcaat-3’	94 °C/45 s	58 °C/45 s	72 °C/1 min	432
		Reverse: 5’-aaagaaagggtgtaaaacgca-3’				
NM031512	IL-1β	Forward: 5’-atgagagcatccagcttcaaatc-3’	94 °C/45 s	58 °C/45 s	72 °C/1 min	328
		Reverse: 5’-gcttatgttctgtccattgaggt -3’				
NM012589	IL-6	Forward: 5’-gacaaagccagagtccttca-3’	94 °C/45 s	58 °C/45 s	72 °C/1 min	229
		Reversed 5’-actaggtttgccgagtagac-3’				
X66539	TNF-α	Forward: 5’-cgagatgtggaactggcaga-3’	94 °C/45 s	58 °C/45 s	72 °C/1 min	256
		Reverse: 5’-ctacgggcttgtcactcga-3’				
NM019178	TLR4	Forward: 5’-gccggaaagttattgtggtg-3’	94 °C/45 s	58 °C/45 s	72 °C/1 min	203
		Reverse: 5’-ccactcgaggtaggtgttt-3’				

Polymerase chain reaction (PCR) amplification was performed with 1 U of Taq DNA polymerase (Promega), 2.5 mM dNTP mix and 1 μM of each primer, using 1 μl of the RT reaction product as template. After amplification, the products were electrophoresised in 2% agarose gel, visualized by ethidium bromide staining and scanned with ultraviolet transilluminator (GDS 8000, Gene Tools from Syngene Software, UK). Bands were analyzed using GeneSnap Image Analysis Software (Syngene, UK). The relative amount of each mRNA was normalized to the housekeeping gene β-actin mRNA, and the data are presented as ratio of the signal intensity of examined gene vs. normal control. To avoid the genomic DNA contamination for RNA preparation, 1 ug of total RNA was used for PCR amplification and no PCR bands were found in the test.

### Multiplex assay for cytokine expression

Four animals of each group were used for cytokine expression by using Bio-Plex analysis. The L4-6 segments of spinal cord were removed from the rats under deep anesthesia with an over dose of sodium pentobarbital. According to the instruction supported by Shanghai Miaotong Bio-Tech. Company, fifty μl of total protein extracted from the lumber enlargement were used for the cytokine analysis. Finally, the cytokine level was read by Bio-Plex 200 System (Bio-Rad Laboratories, Inc).

### Statistical analysis

Data were presented as mean ± S.E.M and were analyzed for statistical significance by one-way analysis of variance (ANOVA) followed by Newman-Keuls test or chi-square test based on necessary, using the Statistical Package for the Social Sciences (SPSS) statistical software. P < 0.05 was considered statistically significant.

## Competing interests

The authors declare that they have no competing interests.

## Authors’ contributions

YQ contributed to the conception and design of the experiments. GC contributed to manuscript drafting and revising. QL, XW and Xiu contributed to the animal behavior, QL and WL contributed to the PCR and Immunohistochemistry. CJ contributed to the Multi-Plex analysis. All authors read and approved the manuscript.
